# Outflow Through Aortic Side Branches Drives False Lumen Patency in Type B Aortic Dissection

**DOI:** 10.3389/fcvm.2021.710603

**Published:** 2021-08-13

**Authors:** Gerlinde Logghe, Bram Trachet, Patrick Segers, Julie De Backer, Joscha Mulorz, Philip Dueppers, Frank Vermassen, Hubert Schelzig, Isabelle Van Herzeele, Markus U. Wagenhäuser

**Affiliations:** ^1^Institute for Biomedical Engineering and Technology, Ghent University, Ghent, Belgium; ^2^Department of Cardiology and Center for Medical Genetics, Ghent University Hospital, Ghent, Belgium; ^3^Department of Vascular- and Endovascular Surgery, Medical Faculty and University Hospital Düsseldorf, Heinrich-Heine-University Düsseldorf, Düsseldorf, Germany; ^4^Department of Vascular Surgery, University Hospital Zurich, University of Zurich, Zurich, Switzerland; ^5^Department of Thoracic and Vascular Surgery, Ghent University Hospital, Ghent, Belgium

**Keywords:** aortic dissection, minor branches, outflow, patency, TEVAR, false lumen, true lumen

## Abstract

**Objective:** Thoracic endovascular aortic repair (TEVAR) for type B aortic dissection (TBAD) aims to induce false lumen (FL) thrombosis by sealing intimal tears between the true (TL) and the FL, and blocking the inflow into the FL. Incomplete thrombosis of the FL is correlated with poor clinical outcome. We hypothesize that the number of major and minor branches arising from the FL affects FL patency and may negatively influence TEVAR induced FL thrombosis.

**Methods:** Computed tomography (CT)-scans from 89 patients diagnosed with TBAD [best medical treatment (BMT) *n* = 52, TEVAR *n* = 37] from two high-volume vascular surgery centers were analyzed retrospectively. Analysis included evaluation of the FL patency status, the number, location and size of intimal tears, and the presence of minor and major side branches originating from the FL. Multiple regression analysis was conducted to evaluate obtained parameters as predictors for FL thrombosis status.

**Results:** In univariate analysis, the strongest correlation for FL patency was found for the number of major (*R* = 0.79) and minor (*R* = 0.86) side branches originating from the FL. When applying a multiple linear regression model, the number of major (normalized beta 0.37; *P* < 0.001) and minor (normalized beta 0.41; *P* < 0.01) side branches arising from the FL were valid predictors for the axial length of the patent and non-patent FL, and additionally determined the length of the patent FL at 12-month follow-up in patients that underwent TEVAR.

**Conclusions:** Our data suggest that the number of minor side branches that originate from the FL in TBAD is an important determinant of FL patency, to a greater degree than previously assumed.

## Introduction

Type B aortic dissection (TBAD) is defined as a tear in the intimal layer of the descending thoracic aorta (DTA) distal to the left subclavian artery (LSA), with consequent blood flow within the medial layer of the aorta (1). Mortality rates vary significantly depending on the acute or chronic nature of the pathology, occurrence of complications and treatment chosen (1–5). Overall pre- and post-discharge mortality and morbidity are high for all types of TBAD, and 5-year patient survival rates range from 50 to 70% (1, 5, 6). Well over 60% of deaths associated with acute TBAD result from aortic rupture, mostly located at the false lumen (FL) (1, 4, 6, 7).

Treatment of TBAD focuses on restoring or maintaining vital organ function and peripheral perfusion while preventing aortic rupture. Uncomplicated TBAD is primarily treated with antihypertensive drugs to prevent progression and aneurysm formation. However, several studies demonstrated an early benefit of thoracic endovascular aortic repair (TEVAR) when compared to antihypertensive therapy alone, although long-term results are pending thorough validation (7–11).

In patients with TBAD complicated by renal, visceral or limb ischemia, persistent pain, refractory hypertension, and rapid or chronic expansion of the aortic diameter open surgical repair (OSR) or TEVAR are often inevitable. The latter aims to seal the primary entry tear and thus reduces blood flow into the FL. By promoting FL thrombosis, TEVAR may also prevent distal extension of the dissection (1). Superior patient outcomes have been reported following TEVAR when compared with OSR (1, 12–14). Despite these benefits, procedure-related complications such as stroke and progression of TBAD may occur (5, 7, 15–18).

It is known that entry tear sizes >10 mm, initial FL diameter and incomplete, partial thrombosis of the FL predict the long-term mortality risk in patients with TBAD (15, 17). Importantly, partial FL thrombosis was shown to be a significant independent predictor of post-discharge mortality (16, 18). Yet, studies exploring predictive factors for partial FL thrombosis are rather scarce. Maximum FL-diameter, (re-) entry tears, blind-sac subtype and large visceral branches arising from the FL have been suggested to influence partial FL thrombosis (7, 19, 20). These parameters relate to the in- and outflow into and from the FL. However, outflow from the FL does not occur only through visceral branches or re-entry tears but may also occur through smaller communicative branches such as intercostal, lumbar and bronchial arteries which may also originate from the FL (21, 22).

In this retrospective exploratory study, we document and quantify the number of major and minor side branches arising from the FL in TBAD and evaluate their role in FL thrombosis. The study is based on the consideration that stasis of blood is a necessary condition to achieve FL thrombosis (23). When an inflow tract into the FL is present [e.g., one or several intimal tears between the true lumen (TL) and the FL], the presence of side branches arising from the FL results in hemodynamic conditions that impede complete stasis of the blood. In this context, previous observations from our group in Angiotensin-II—infused mice, a popular mouse model for aortic dissection (24–26), suggested an important role of both minor and major side branches in FL thrombosis and disease progression (26, 27).

The aim of the present study is to test the hypothesis that outflow from the FL through major and minor side branches affects FL patency by providing a continuous hemodynamic connection between the TL and FL.

## Methods

### Data Collection

Medical records and vascular imaging data of patients suffering from TBAD who were treated in the Department of Vascular and Endovascular Surgery at the University Hospital Düsseldorf (UKD) and in the Department of Thoracic and Vascular Surgery at Ghent University Hospital (UZG) were screened retrospectively following approval from the local ethics committess (study ID: UKD: 2017064325; UZG: EC/2017/1635). Patients were excluded from the study if they (i) did not agree to participate, (ii) suffered from known genetic disorders, (iii) had a (history of) type A aortic dissection, (iv) were treated with OSR or (v) had poorly contrasted CT angiograms.

Baseline was defined by the first available Computed tomography (CT)-scan after admission. Demographic data (sex and age) and risk factors (pre-existing arterial hypertension, active smoking, diabetic status, statins on admission, family history of aortic dissection, and renal insufficiency) were obtained for all patients. In patients undergoing TEVAR, indications for intervention, stent graft information (number of stent grafts, coverage length) and complications during or following surgery (spinal cord ischemia, transient ischemic attack (TIA), stroke or death) were evaluated.

### Image Analysis

**Figure 1:**
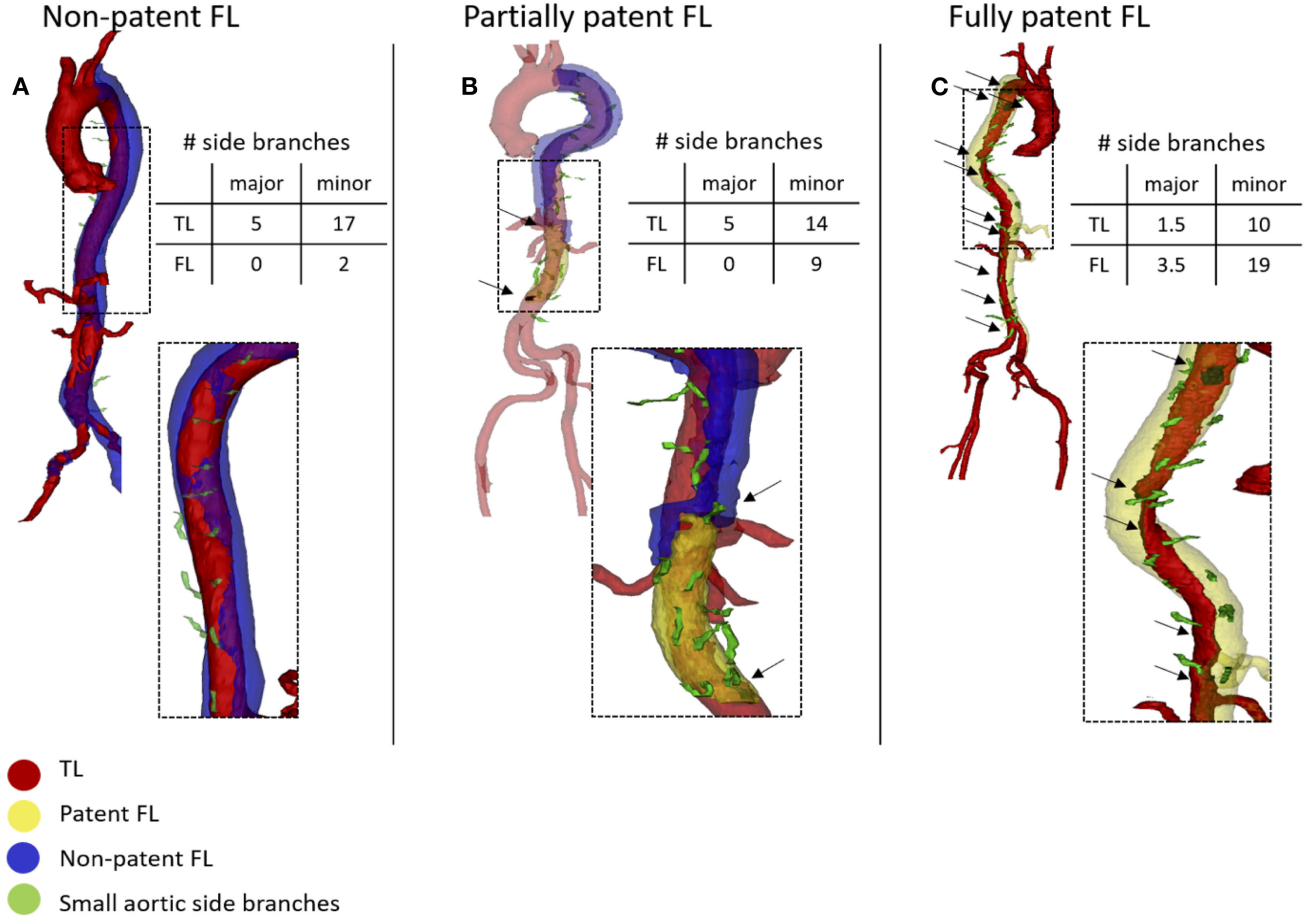
3D segmentation of three representative baseline Computed-tomography (CT)-scans. Black arrows indicate intimal tears. The false lumen (FL) is non-patent along its entire length **(A)**. Non-patent FL in the proximal segment, partially patent FL at the level of major visceral branches, fully patent FL in the distal segment **(B)**. Fully patent FL along its entire length **(C)**. Minor branches originate from the FL in fully patent segments of the FL, while they originate from the TL in non-patent segments of the FL **(A)**. In partially patent FL **(B)** minor branches originate from the FL in the patent segment and from the TL in the non-patent segment.

CT images from patients from the UZG and UKD databases were acquired using state-of-the-art clinical scanners from different manufacturers (Siemens, Erlangen, Germany; Toshiba, Tokyo, Japan; Phillips Healthcare, Hamburg, Germany; Canon Medical, Otawara, Japan). Slice thickness varied from 1 to 5 mm for the UZG patients, and from 0.75 to 5 mm for the UKD patients with 5 mm slice thickness CT-scans in 3 patients across both institutions. Image analysis was performed using Mimics 19 (Materialize, Leuven, Belgium) and similarly applied to all datasets. Initially, the aorta was cropped from the first slice distal to the LSA down to the last axial slice above the aortic bifurcation. Within this region of interest (ROI), the aortic domain was subdivided into three different zones: TL, patent FL and non-patent FL. Figure 1 shows three representative and segmented aortae with each of the three aforementioned zones indicated by a different color. The distinction between patent and non-patent FL was based on differences in mean gray scale values. We then quantified the number of major side branches (celiac, superior and inferior mesenteric, left and right renal arteries) and minor side branches (intercostal, lumbar, gonadal arteries, middle sacral artery) arising from the TL, the patent and non-patent FL. Each additional branch resulted in an addition of 1.0 to the cumulative score. For branches that originate from both the TL and the FL, a score value of 0.5 was added.

Each axial CT slice within the ROI was subsequently assigned one of four patency labels: no FL, fully patent FL, non-patent FL or partially patent FL (when a segment of the FL was patent and another segment was non-patent) (Figure 1). The axial length of the TL, patent FL, and non-patent FL was calculated as the number of axial slices that bore the corresponding patency label, multiplied with the interaxial slice distance, which was constant for each individual scan. For the calculation of axial lengths, the patent FL was defined to include all slices with either a fully patent or a partially patent FL. The axial distance of the non-patent FL was hence based on axial CT slices in which the entire cross-section of the FL was non-patent. The relative FL cross-sectional area was quantified as the ratio of the local 2D FL lumen area surface over the complete lumen area (TL + FL).

In a next step, we quantified the number and the volume of intimal tears within the ROI. An intimal tear was defined as a disruption of the intimal flap separating the TL and FL. Intimal tears were counted if a disruption was visible on at least two of three views (axial, sagittal and/or coronal view). A disruption was attributed a score value of 0.5, if it was only visible on one of the three views, or not clearly visible on several views. The volume of an intimal tear was defined as the volume of the tissue missing from the aortic wall at the location of the intimal tear. In order to quantify the volume of the intimal tear, the thickness of the missing tissue was assumed in correspondence to the surrounding aortic wall. The volume of intimal tears was semi-automatically quantified in 3D using Mimics. The axial distance between the LSA and the most proximal slice of the most proximal intimal tear was estimated based on the number of axial slices between both landmarks.

### Analysis of Baseline Data

Rstudio (Version 1.3.959, © 2009-2020 RStudio, Public Benefit Corporation) was used for statistical analyses. Mann-Whitney-Wilcoxon Test was used to evaluate differences between the two institutions for the quantified parameters (ns: *p* > 0.05, ^**^p ≤ 0.01). Spearman's rank correlation coefficient was used to examine the correlation between the axial length of the patent segment of the FL and other parameters. Kruskal-Wallis test was used to compare the axial length of different patency zones (patent FL, non-patent FL, no FL) between the different institutions (UZG vs. UKD). Multiple regression analysis was performed for the axial length of the patent portion of the FL as dependent variable and the number and size of intimal tears, the distance from the LSA to the most proximal intimal tear, the relative FL area, the number of major side branches and the number of minor side branches arising from the FL, and the number of minor side branches originating from the TL as independent variables. A similar model was set up with the axial length of the non-patent portion of the FL as dependent variable. Results are presented as forest-plots of standardized beta values (dividing by two standard deviations). *P*-values ≤ 0.05 were considered statistically significant.

### Analysis of Follow-Up Data

Similar to the baseline data, the axial length of the fully patent, partially patent and non-patent FL segments of the aorta were determined, as well as of the non-dissected aorta between the LSA and the aortic bifurcation. We assessed the correlation between the axial length of the patent FL and its assumed determinants in univariate analysis and multiple linear regression models. We also compared the axial length of the patency status in the FL and the non-dissected lumen between the two treatment groups (BMT, TEVAR). For this comparative analysis, lengths were normalized to the total length of the aortic segment from the LSA to the aortic bifurcation.

## Results

### Baseline Data

A total of 89 patients was included in this study. Given the retrospective study design, follow-up intervals varied. Follow-up CT-scans of patients receiving best medical treatment (BMT) were binned into five different time points: baseline (*n* = 89), 2–4 months (*n* = 41), 10–14 months (*n* = 16), 20–28 months (*n* = 15), 40–56 months (*n* = 4) after baseline (Supplementary Figure 1, upper panel). Of these, 37 patients underwent TEVAR, with a mean period from baseline to TEVAR of 126 ± 191 days. The last CT-scan prior to intervention was considered as TEVAR baseline. Subsequent follow-up scans were binned relative to TEVAR baseline, i.e., 2–4 months (*n* = 17), 10–14 months (*n* = 21), 20–28 months (*n* = 17), and 40–56 months (*n* = 10) following surgery (Supplementary Figure 1, lower panel). Of note, there was no spinal cord ischemia following TEVAR. Baseline demographics and risk factors are displayed in Table 1. Mean follow-up for BMT and TEVAR cohorts was 320 ± 486 days and 993 ± 772 days, respectively.

**Table 1:** Age, sex and risk factors on admission are displayed for all patients at baseline.

**Patients at baseline (** ***n*** **=** **89)**	
**Age in years**	
	65.2 (13.6)
**Sex**	
Male/female	72/17 (80.9%/19.1%)
Risk Factors on admission^*^	
Pre-existing arterial hypertension	78 (87.6%)
Active smoking	35 (39.3%)
Diabetic	12 (13.5%)
Statin treatment	23 (25.8%)
Family history of aortic dissection	10 (11.2%)
Renal insufficiency	12 (13.5%)
**Patients undergoing TEVAR (** ***n*** **=** **37)**	
**Indications for intervention**	
Refractory pain/uncontrolled hypertension	8 (21.6%)
Malperfusion	8 (21.6%)
Rapid enlargement of the aorta	11 (29.7%)
Rupture	0 (0%)
Change of disease class/type	7 (18.9%)
Other/unknown e.g., surgeon's preference,.	7 (18.9%)
**Number of stent grafts**	
	1.14 (0.419)
**Lenght of aortic coverage (mm)**	
	150 (53.6)
**Complications following surgery**	
Spinal cord ischemia	0 (0%)
TIA/stroke	0 (0%)
Death	2 (5.4%)

*For patients that received TEVAR treatment, indications for intervention, number of implanted stent grafts, length of aortic coverage and complications following surgery are displayed. Results are presented as absolute frequency n (%) or mean with (standard deviation)*.

* *Risk factors as reported in the medical records of the patients. TEVAR, Thoracic endovascular aortic repair; TIA, transient ischemic attack*.

### Functional Parameters

The distribution of the quantified parameters for both hospitals is provided in Supplementary Figure 2. There were no significant differences between the two patient collectives, except for the average number of intimal tears/patient (3.6 ± 3.24 tears/patient for UKD; 1.65 ± 1.75 tears/patent for UZG, Mann-Whitney-Wilcoxon, *p* ≤ 0.01).

**Figure 2:**
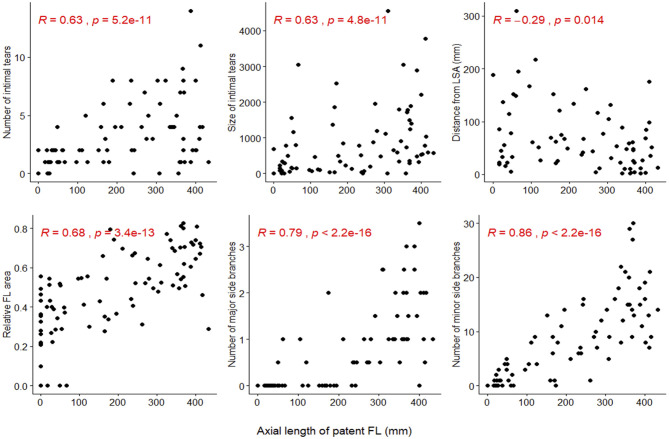
Correlation between the axial length of the patent false lumen (FL) (x-axis) and the number and size of intimal tears, the distance from the left subclavian artery (LSA) to the most proximal intimal tear (distance from LSA), the relative FL area, and the number of major and minor side branches arising from the FL (y-axis). Spearman's rank correlation was applied and the corresponding *p*-value is displayed for each scatter-plot.

The number of major (*R* = 0.79) and minor (*R* = 0.86) side branches arising from the FL showed the highest correlation with the axial length of the patent FL in univariate analysis (Figure 2). Other quantified parameters, such as the number (*R* = 0.63) and size (*R* = 0.63) of intimal tears, the distance from the most proximal intimal tear to the LSA (*R* = −0.29), and the relative FL area (*R* = 0.68) correlated less strongly with FL patency, although all correlation coefficients reached statistical significance.

### Determinants of False Lumen Patency

**Figure 3:**
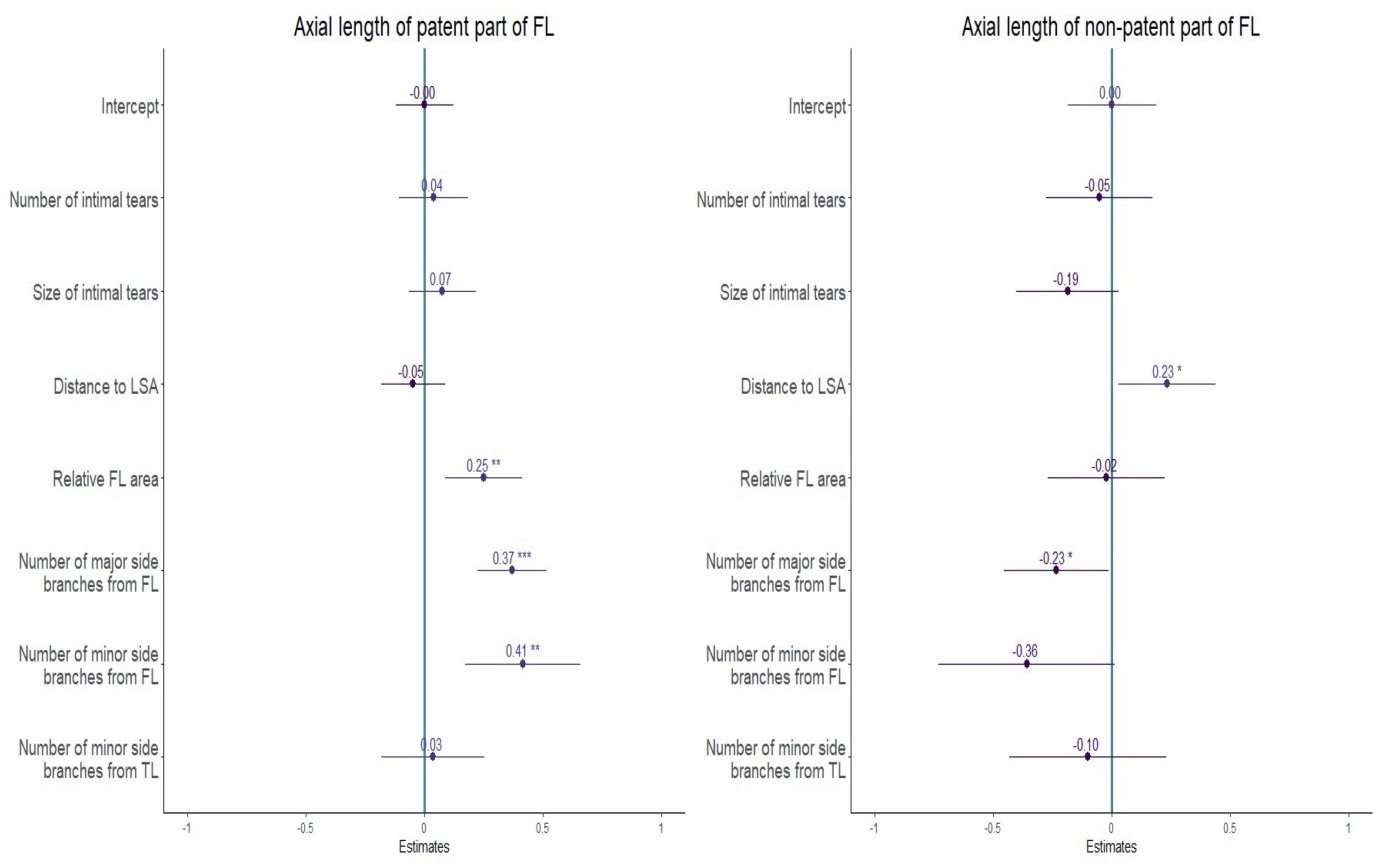
Forest-plot of standardized beta values in a multiple regression model for the axial length of the patent false lumen (FL) (left) and the non-patent FL (right) at baseline. The dependent variables are the number and size of the intimal tears, the distance from the first intimal tear to the left subclavian artery (LSA) (distance to LSA), the relative FL area, the number of major and minor side branches arising from the FL, and the number of minor side branches arising from the true lumen (TL). Standardized by division of two standard deviations. *p ≤ 0.05; **p ≤ 0.01, ***p ≤ 0.001. The resulting multiple linear regression model predicted the length of the patent FL with good accuracy (*R*^2^ = 0.77), while the prediction was somewhat lower for the non-patent FL (*R*^2^ = 0.47).

The number of minor (standardized beta 0.41, *P* < 0.01) and major side branches (standardized beta 0.37, *P* < 0.001) originating from the FL emanated as the strongest determinants of the regression model to predict the axial length of the patent portion of the FL in multiple linear regression analysis (Figure 3). The relative FL area had a (minor) positive effect (normalized beta 0.25, *p* ≤ 0.01) in the patent FL regression model. When predicting the axial length of the non-patent portion of the FL in the regression model, the distance from the first intimal tear to the LSA was the only parameter with a (minor) positive effect (normalized beta 0.23, *p* ≤ 0.05). The number of major branches arising from the FL, as well as the number of minor branches arising from the FL, had minor negative effects in this model (normalized beta −0.23, *p* ≤ 0.05 and normalized beta −0.36, *p* > 0.05, respectively). In both, the patent and non-patent FL regression model, the number and size of the intimal tears and the number of minor branches originating from the TL only marginally contribute to the prediction (Figure 3).

### Analysis of Follow-Up Data

**Figure 4:**
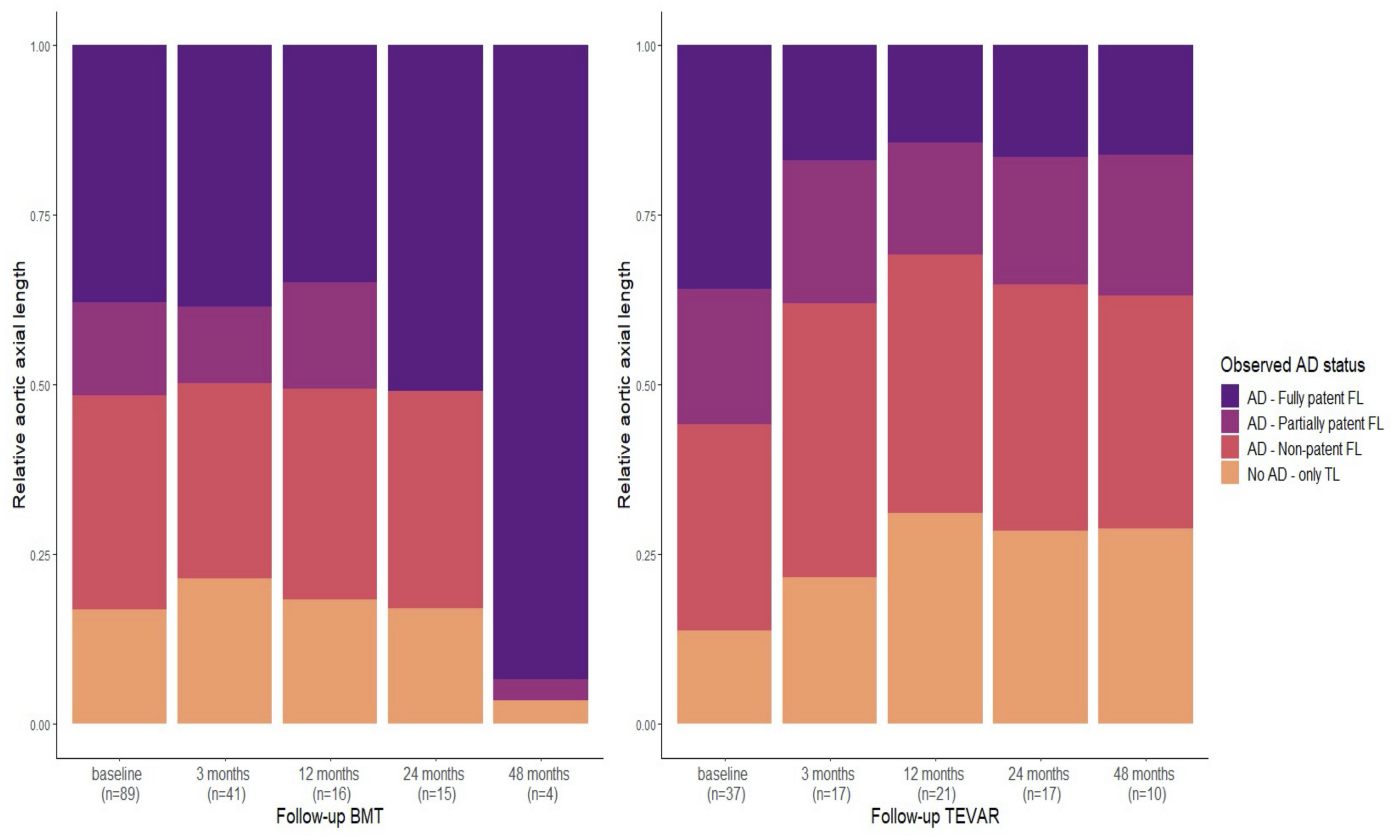
Relative mean axial length of the aorta following assignement to one of the four patency status [fully patent false lumen (FL), partially patent FL, non-patent FL, and no aortic dissection (AD)—only true lumen (TL)] in patients with type B aortic dissection (TBAD) treated either by best medical treatment (BMT) or subsequent thoracic endovascular aortic repair (TEVAR) at 3, 12, 24, and 48 months follow-up (see also Supplementary Figure 1). “No AD—only TL” indicates the portion of the aorta where no AD/FL was observed (anymore). Note that TEVAR treatment reduces the relative portion of the fully patent FL, while the same is not true for the partially patent FL.

The portion (= “relative axial length”) of the fully patent FL was lower for patients undergoing TEVAR when compared to patients receiving BMT (Figure 4) at all follow-up time points (*p* < 0.05). However, the relative length of the partially patent FL remained unchanged following TEVAR treatment (*p* > 0.05 in all TEVAR follow-up groups). Also, no significant difference in relative length of the partially patent FL was found between the BMT vs. TEVAR group at all follow-up time points (*p* > 0.05).

**Figure 5:**
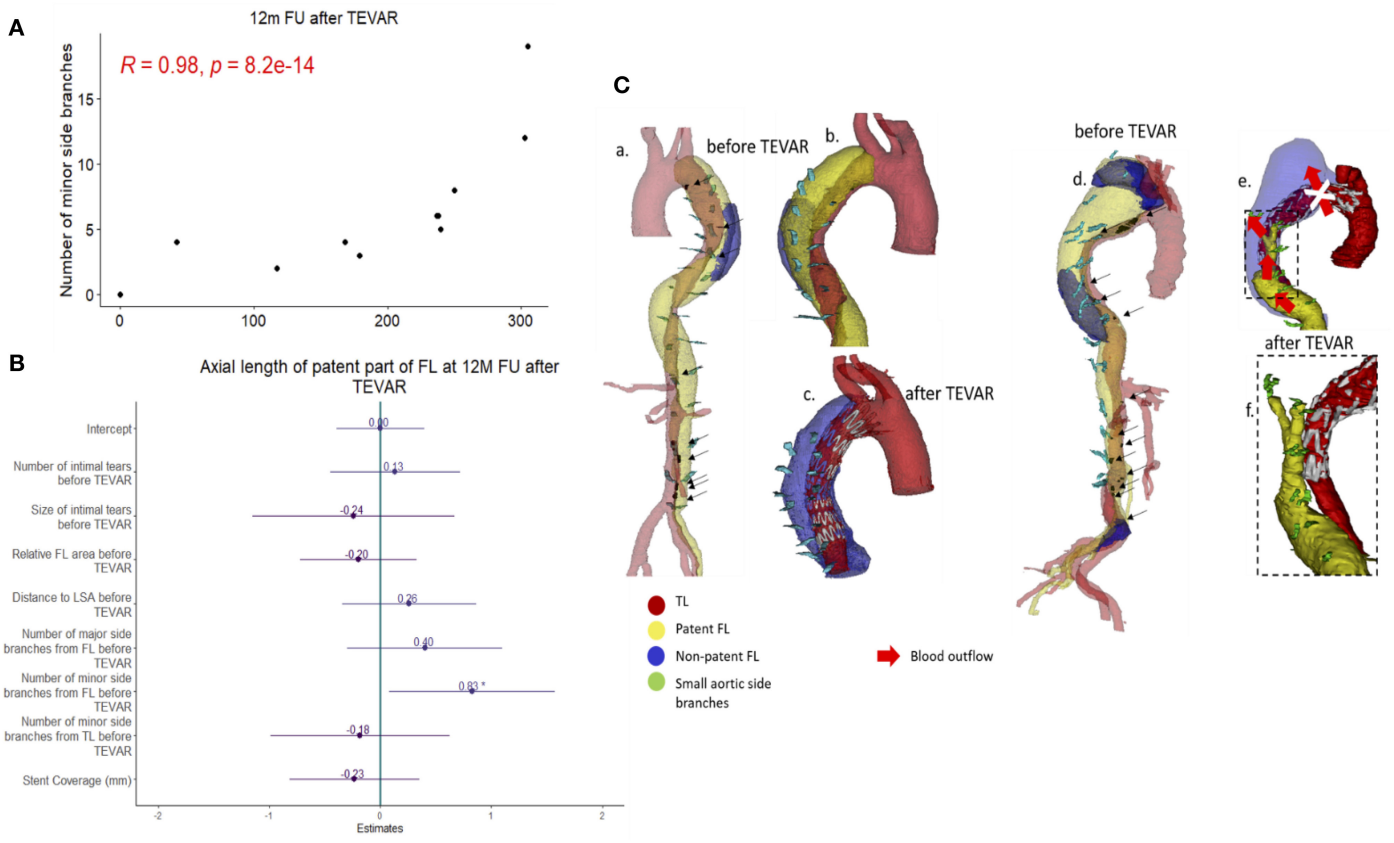
Correlation between the patent false lumen (FL) length and the number of minor side branches arising from the FL **(A)** Spearman's rank correlation was applied and the corresponding *p*-value is displayed. Forest-plot of standardized beta values for a multiple linear regression model for the axial length of the patent FL at 12 months follow-up for patients receiving thoracic endovascular repair (TEVAR) **(B)**. Two cases of aortic remodeling following TEVAR **(C)**. For the case on the left, the FL is partially patent prior to TEVAR (a,b) and non-patent following TEVAR (c). For the case on the right, the FL is partially patent both prior to (d) and following (e,f) TEVAR. Note that partial retrograde patency of the FL occurs which sustains blood flow to minor side branches arising from the FL prior to TEVAR.

Most data for TEVAR were available at 12 months follow-up (*n* = 21). There was a significant correlation between the patent FL length and the number of minor side branches arising from the FL in univariate analysis (Figure 5A) and multivariate linear regression analysis (Figure 5B; normalized beta 0.83, *p* < 0.05). Data from other time points are provided in Supplementary Figure 3. In univariate analysis, a positive and significant relation was found between the patent FL length and the number of minor FL side branches at all time points.

## Discussion

**Figure 6:**
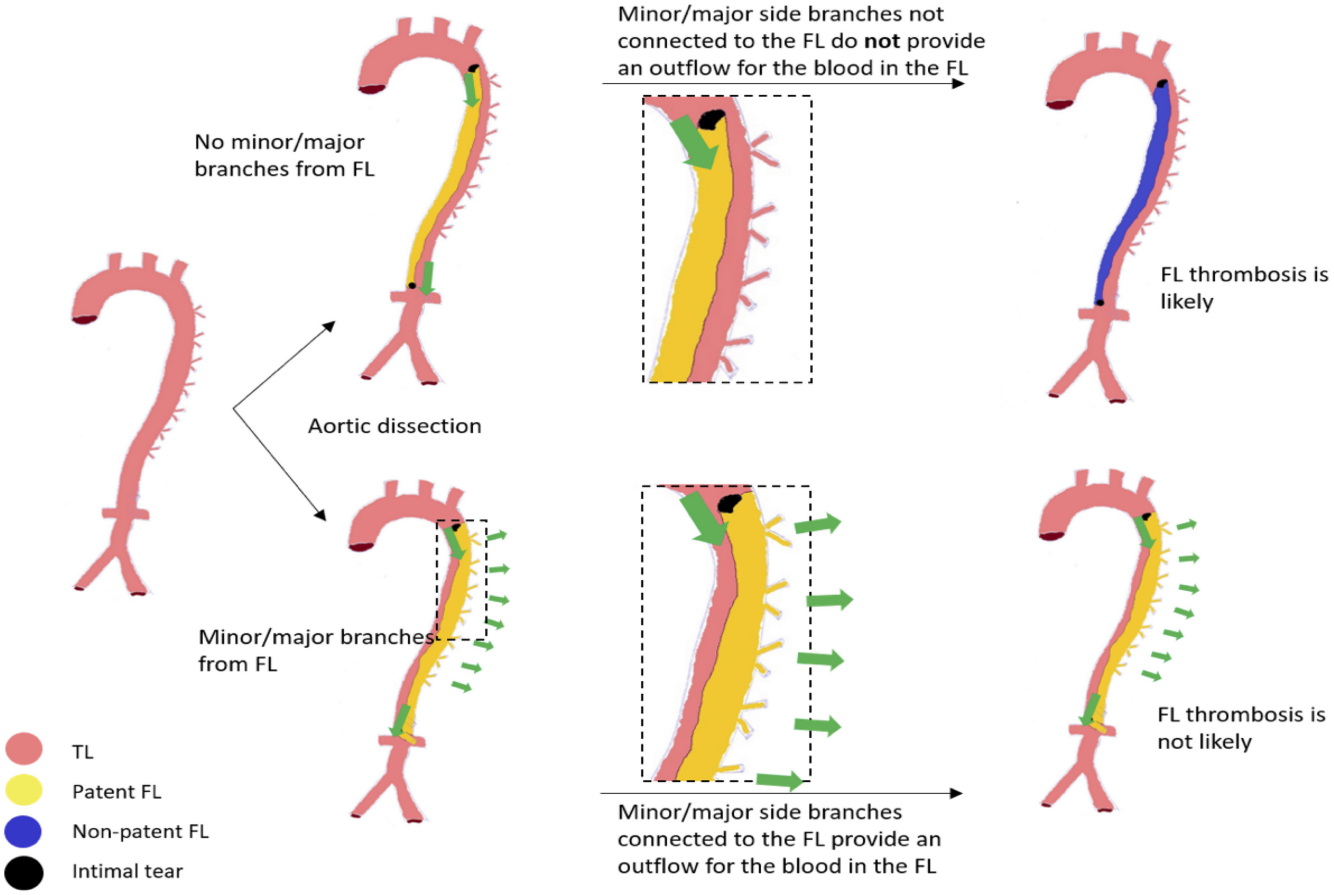
Schematic illustration of the proposed mechanism determining false lumen (FL) patency driven by small side branches. Intimal tears are black, while green arrows indicate FL blood flow. Left: healthy aorta with no tears and no aortic dissection (AD). At the time of AD, a FL and true lumen (TL) is created through delamination of the aortic wall. Note that more than one intimal tear may occur. Upper panel: No side branches originate from the FL; all minor side branches originate from the TL. Here, the outflow is limited *via* re-entry tears. Stasis of blood flow is likely which might initiate FL thrombosis. Lower panel: (Multiple) Minor side branches originate from the FL and provide a continuous outflow for the blood. FL thrombosis is less likely to be initiated.

At the onset of this study, we hypothesized that in patients with TBAD, outflow through major and minor branches arising from the FL maintains blood flow through the FL and thus prevents FL thrombosis. The data gathered in this retrospective study strongly supports this hypothesis. Both univariate and multiple regression analysis identified the number of branches originating from the FL as the most important predictor for lumen patency. Despite the relatively small number of TEVAR cases, follow-up data further suggest that FL side branches remain a major determinant of FL patency in patients following thoracic stent graft placement. Based on these results we advocate that FL thrombosis is unlikely to be achieved in the presence of (minor) side branches arising from a perfused FL (Figure 6).

It is important to emphasize that our investigation did not study nor question the importance of intimal tears for FL patency and patient outcomes in TBAD (17). Indeed, for an outflow to occur from the FL, inflow needs to be provided by at least one intimal tear connecting the TL and FL and our data confirm the significance of tear size and location for FL patency. However, these inflow parameters appear less significant when compared to outflow parameters such as the number of branches arising from the FL when applying multiple linear regression models.

Since minor FL branches might sustain the circulation of blood within (parts of) the FL, we further hypothesized that TEVAR treatment is less likely to induce FL thrombosis in case (i) side branches originate from the FL and (ii) blood flow into the FL is still present following TEVAR. Successful treatment by inducing FL thrombosis may therefore depend on the number of branches arising from the FL. This hypothesis seems in line with patient outcomes following TEVAR, which reported complete FL thrombosis in up to 40% of all cases while persistent distal perfusion may lead to late FL expansion in up to 30% of all cases during follow-up (28, 29).

Our data support the aforementioned hypothesis. TEVAR did not reduce the axial length of partial thrombosis (Figure 4). The proposed underlying mechanism is illustrated in Figure 5 (right panel) on 3D segmented aortae for two exemplary cases. Since TEVAR may only block FL inflow at the proximal intimal entry tear, outflow through side branches arising from the FL is present if tears distal to the implanted stent graft (so-called re-entry tears) are not blocked. This leads to retrograde flow into the FL with inflow from distal tears and outflow into small side branches. In these cases, blocking the inflow from the primary entry tear by TEVAR does not result in a fully thrombosed FL but predisposes for a partially thrombosed FL with residual flow into the FL from distal tears and outflow into minor side branches. This concept is also supported by our, albeit slightly underpowered, follow-up study. Despite the low number of TEVAR follow-up cases, the follow-up data at 12 months demonstrated a significant correlation between the extent of patent FL and the number of side branches arising from the FL. The number of side branches originating from the FL was the only variable reaching statistical significance in multiple regression analysis in TEVAR patients at 12 months follow-up (Figure 5). It is important to emphasize that these observations do not question the efficacy of TEVAR treatment in general. In fact, our data support the success of TEVAR in promoting FL thrombosis. Patients treated with TEVAR had increased relative axial length of the non-patent FL when compared to those receiving BMT, which was true for all patients at all follow-up time points.

Over time, several techniques have been introduced to enhance FL thrombosis and aortic remodeling following TEVAR. Extended aortic covering utilizing longer stent grafts were found to bear a higher risk of spinal cord ischemia while seeking to occlude multiple tears between the TL and FL aiming to reduce pressure and flow transmission into the FL (30, 31). The so-called Knickerbocker Technique is based on the dilation of the central stent graft segment in the TL to rupture the dissection membrane aiming to punctually compress the FL (32). Other authors used FL lumen access for plug (so-called candy-plug), coil or glue deployment to block retrograde inflow and promote FL thrombosis (33, 34). While all these approaches aim to reduce inflow into the FL, our data advocate that controlling the outflow might be equally important. This is also supported by findings of the ADSORB trial, which noted that the number of vessels originating from the FL may predict FL growth in patients with TBAD (35). Taking this into consideration, specifically targeting side branches that originate from the FL aiming to block the outflow from the FL seems beneficial and feasible by e.g., selective embolization, since such procedures have already been described and demonstrated an effective induction of FL thrombosis in TBAD while avoiding device deployment into the FL (36). Such device deployment into the FL might hold several risks such as narrowing of the TL, damaging the dissection membrane, device migration and breakage or endoleakage. In contrast, a more specific approach aiming to occlude originating FL branches (e.g., *via* embolization) would minimize the amount of implanted material and device deployment-associated risks (37).

While the association between (partial) FL patency and survival rates in TBAD has been studied extensively (28, 38–41), little is known about the underlying and causal mechanisms. It has been hypothesized that pulsatile inflow through intimal tears into a lumen with impaired outflow (when there are no re-entry tears) increases blood pressure in such FL, which in turn subsequently leads to a higher risk of lumen expansion, dissection progression and rupture (13, 16, 40, 41). However, the direct effect of inflow on FL pressure is expected to be limited while maximally arising to the dynamic pressure, which would equal <5 mmHg when assuming a peak aortic velocity of ~1 m/s (estimated from the simplified Bernoulli equation with dynamic pressure, in mmHg, approximating 4v^2^ with v the velocity in m/s). Moreover, this hypothesis does not consider that branches arising from the FL provide an alternative outflow, thus preventing pressure build-up within the FL. From a hemodynamic perspective, the alternative hypothesis we propose here provides an elegant solution for these problems.

Our study has several limitations. First, only two centers retrospectively collected patient data, thus limiting cohort sizes. Next, high-resolution CT images are indispensable to properly identify minor side branches and intimal tears. Intimal tears can sometimes be mistaken for a flapping aorta, while the smallest side branches sometimes take up only few pixels on a single cross-section. All quantifications were performed directly on the images by two well-trained observers (BT and GL). Nevertheless, variations due to interpretation errors cannot be excluded, also given that there was a difference in mean slice thickness between UZG (2.26 ± 0.83 mm) and UKD (1.4 ± 1.08 mm). The latter may explain why UZG patients had significantly fewer intimal tears when compared with UKD patients, although there was no significant difference in the total number of detected minor side branches. Also, minor side branches arising from a thrombosed FL receive proportionally less blood flow and are thus more difficult to detect on CT images compared to minor side branches that arise from a patent FL. This may have affected the results of our study. However, we did not observe significant differences (Wilcoxon test *p* > 0.05) in the total number of minor side branches between patients with a fully thrombosed (22.5 ± 4.22) and patients with a fully patent FL (24.1 ± 3.98). Since FL patency was determined based on differences in gray scale values, the timing of the CT-scan following contrast-bolus injection could have also affected the assessed FL patency. Finally, we approximated the axial length of the patent and the non-patent FL by multiplying the number of axial CT slices on which either was visible with the distance between two slices. In this regard, using 3D volumes instead of axial lengths, which is more labor intensive, could increase accuracy, since it would allow us to take the shape of the aorta also into account.

Potential clinical implications of the findings have not been in the scope of this exploratory study; however, generating a clinical score based on the number of arising minor branches from the FL (TL) and locations/sizes of intimal tears *via* smart computer algorithms to provide an estimation on whether the aorta is at risk for expansion or even whether TEVAR may be beneficial in terms of FL thrombosis induction seems generally feasible at long-term. Although potentially valuable, a cut-off value for such score needs to be defined and validated first and would further require correlation with clinical and morphological outcomes to prove its effectiveness in a preferably prospective setting. Also, potential alternative approaches, such as the specific occlusion of originating FL side branches *via* endovascular procedures needs thorough assessments of its feasibility in challenging anatomies. From a technical point of view, standardized imaging criteria may have to be set, and image processing tools (ideally based on artificial intelligence to favor automation) will have to be developed and validated for a time-efficient and objective quantification of (minor) side branches. Ideally, potential future clinical studies may be complemented with fundamental (bioengineering) research, to provide insights on how false lumen patency is (biomechanically) linked to patient outcome and disease progression. Here, realistic computational fluid-structure interaction models accounting for in- and outflow of the FL *via* intimal tears and side branches may help to elucidate the pressure built-up within the FL and mechanical stresses within the aortic wall. Of note, such models may also consider thrombosis (42, 43).

In conclusion, we have demonstrated that (minor) side branches that originate from the FL in TBAD patients are closely correlated with FL patency. We hypothesize that this is due to outflow from the FL into the branches. Our data advocate that controlling the outflow might be equally important to controlling the inflow in the treatment of patients with TBAD.

## Data Availability Statement

The raw data supporting the conclusions of this article will be made available by the authors, without undue reservation.

## Ethics Statement

The studies involving human participants were reviewed and approved by Ethic committee of the Medical Faculty at the University Hospital Düsseldorf, Germany (ID: 2017064325) and the Ethic Committee at the Univeristy Hospital Ghent, Belgium (EC/2017/1635). Written informed consent for participation was not required for this study in accordance with the national legislation and the institutional requirements.

## Author Contributions

GL, BT, PS, HS, and MUW designed the study. JDB, JM, PD, FV, and IVH acquired the data. GL performed the computation. BT, PS, IVH, and MUW supervised the progress of the study. GL, BT, PS, JM, HS, and MUW analyzed and interpretated the data and drafted the manuscript. JDB, PD, FV, and IVH revised the manuscript for critical intellectual content. All authors approved the final version of the manuscript.

## Conflict of Interest

The authors declare that the research was conducted in the absence of any commercial or financial relationships that could be construed as a potential conflict of interest.

## Publisher's Note

All claims expressed in this article are solely those of the authors and do not necessarily represent those of their affiliated organizations, or those of the publisher, the editors and the reviewers. Any product that may be evaluated in this article, or claim that may be made by its manufacturer, is not guaranteed or endorsed by the publisher.

## Supplementary Material

Supplementary Figure 1 Overview of the number of Computed tomography (CT)-scans included in this study. Each dot represents a CT-scan. The x-axis displays the time interval from the baseline CT-scan (upper panel) or from the time of thoracic endovascular repair (TEVAR) placement (lower panel) in months. A random value was used on the vertical axis to allow for the dots to be dispersed. At onset, all 89 patients were in the best medical treatment (BMT) group and received a baseline CT-scan. Of these, 37 patients underwent TEVAR during the further follow-up and were included in the sub-study. Here, the last CT-scan prior to TEVAR was considered as TEVAR baseline.

Supplementary Figure 2 Distribution of the number and size of intimal tears, the distance from the first intimal tear to the left subclavian artery (LSA), the relative false lumen (FL) area, and the number of major and minor side branches originating from the FL across the two specialized Vascular Surgery Departments. UKD: Department of Vascular and Endovascular Surgery at the University Hospital Düsseldorf, Germany; UZG: Department of Thoracic and Vascular Surgery at the University Hospital Ghent, Belgium.

Supplementary Figure 3 Upper row: Correlation between the patent false lumen (FL) length and the number of minor side branches originating from the FL at 3-, 24-, and 48-months follow-up for patients receiving thoracic endovascular repair (TEVAR). Spearman's rank correlation was applied, and the corresponding *p*-value is displayed. Lower row: Forest-plot of standardized beta values for a multiple linear regression model of the axial length of the patent FL for the same time points. At 48 months follow-up, the model matrix was rank deficient and stent graft coverage was not estimable (also see Figure 5 for 12 months follow-up).
