# Supplementary figures and images for: Outflow Through Aortic Side Branches Drives False Lumen Patency in Type B Aortic Dissection
Source: Front Cardiovasc Med. 2021 Aug 13;8:710603. doi: 10.3389/fcvm.2021.710603 (PMC8414589; doi:10.3389/fcvm.2021.710603)

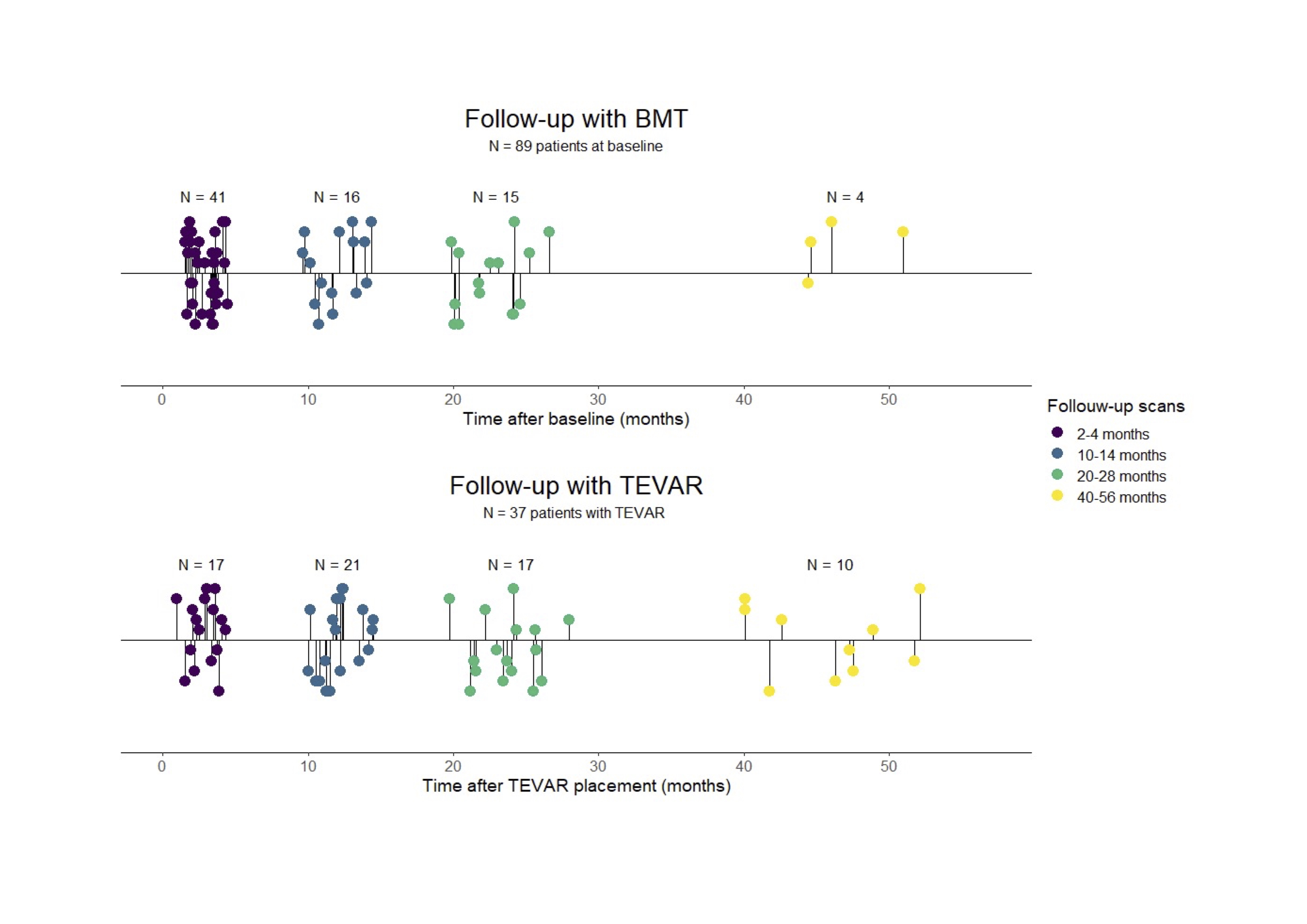

Supplement: Supplementary Figure 1 — Overview of the number of Computed tomography (CT)-scans included in this study. Each dot represents a CT-scan. The x-axis displays the time interval from the baseline CT-scan (upper panel) or from the time of thoracic endovascular repair (TEVAR) placement (lower panel) in months. A random value was used on the vertical axis to allow for the dots to be dispersed. At onset, all 89 patients were in the best medical treatment (BMT) group and received a baseline CT-scan. Of these, 37 patients underwent TEVAR during the further follow-up and were included in the sub-study. Here, the last CT-scan prior to TEVAR was considered as TEVAR baseline. [file Image_1.JPEG]

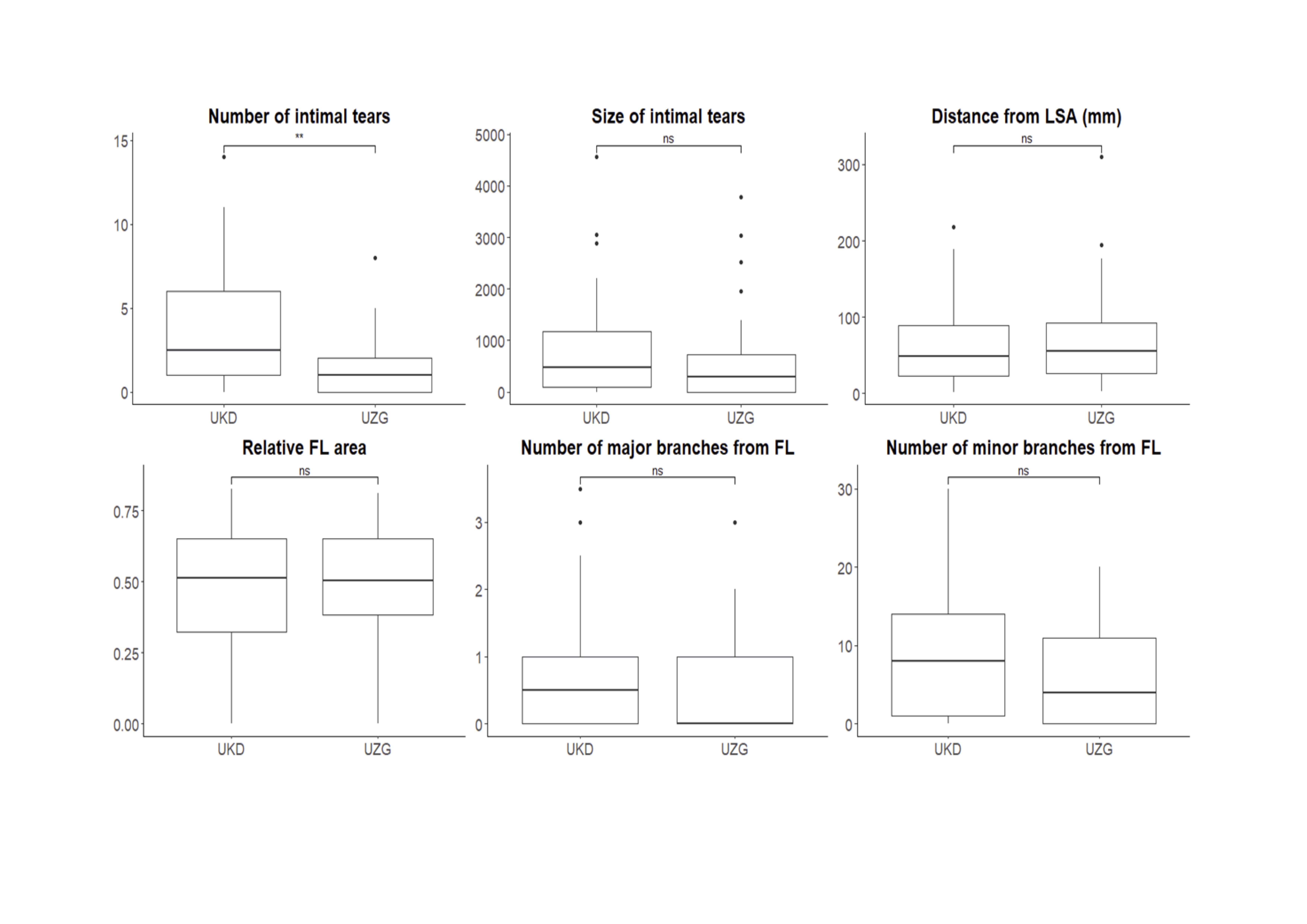

Supplement: Supplementary Figure 2 — Distribution of the number and size of intimal tears, the distance from the first intimal tear to the left subclavian artery (LSA), the relative false lumen (FL) area, and the number of major and minor side branches originating from the FL across the two specialized Vascular Surgery Departments. UKD: Department of Vascular and Endovascular Surgery at the University Hospital Düsseldorf, Germany; UZG: Department of Thoracic and Vascular Surgery at the University Hospital Ghent, Belgium. [file Image_2.JPEG]

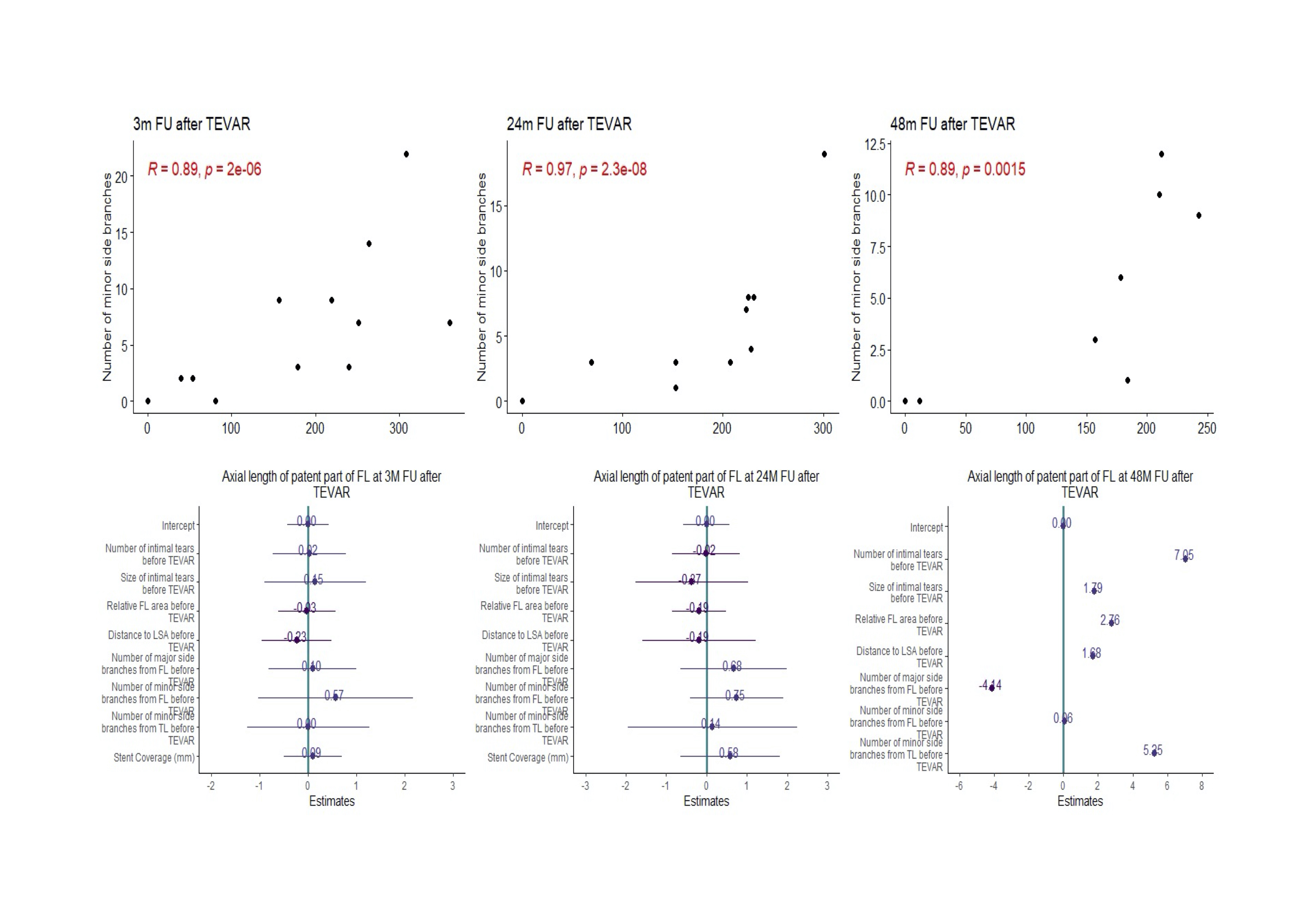

Supplement: Supplementary Figure 3 — Upper row: Correlation between the patent false lumen (FL) length and the number of minor side branches originating from the FL at 3-, 24-, and 48-months follow-up for patients receiving thoracic endovascular repair (TEVAR). Spearman's rank correlation was applied, and the corresponding p-value is displayed. Lower row: Forest-plot of standardized beta values for a multiple linear regression model of the axial length of the patent FL for the same time points. At 48 months follow-up, the model matrix was rank deficient and stent graft coverage was not estimable (also see Figure 5 for 12 months follow-up). [file Image_3.JPEG]
